# Supplementary material for: Candle Soot as a Novel Support for Nickel Nanoparticles in the Electrocatalytic Ethanol Oxidation
Source: Nanomaterials (Basel). 2024 Jun 18;14(12):1042. doi: 10.3390/nano14121042 (PMC11206670; doi:10.3390/nano14121042)
Supplement: Supplementary file 1 [file nanomaterials-14-01042-s001.zip › nanomaterials-3033530-supplementary.pdf]

Supplementary Materials

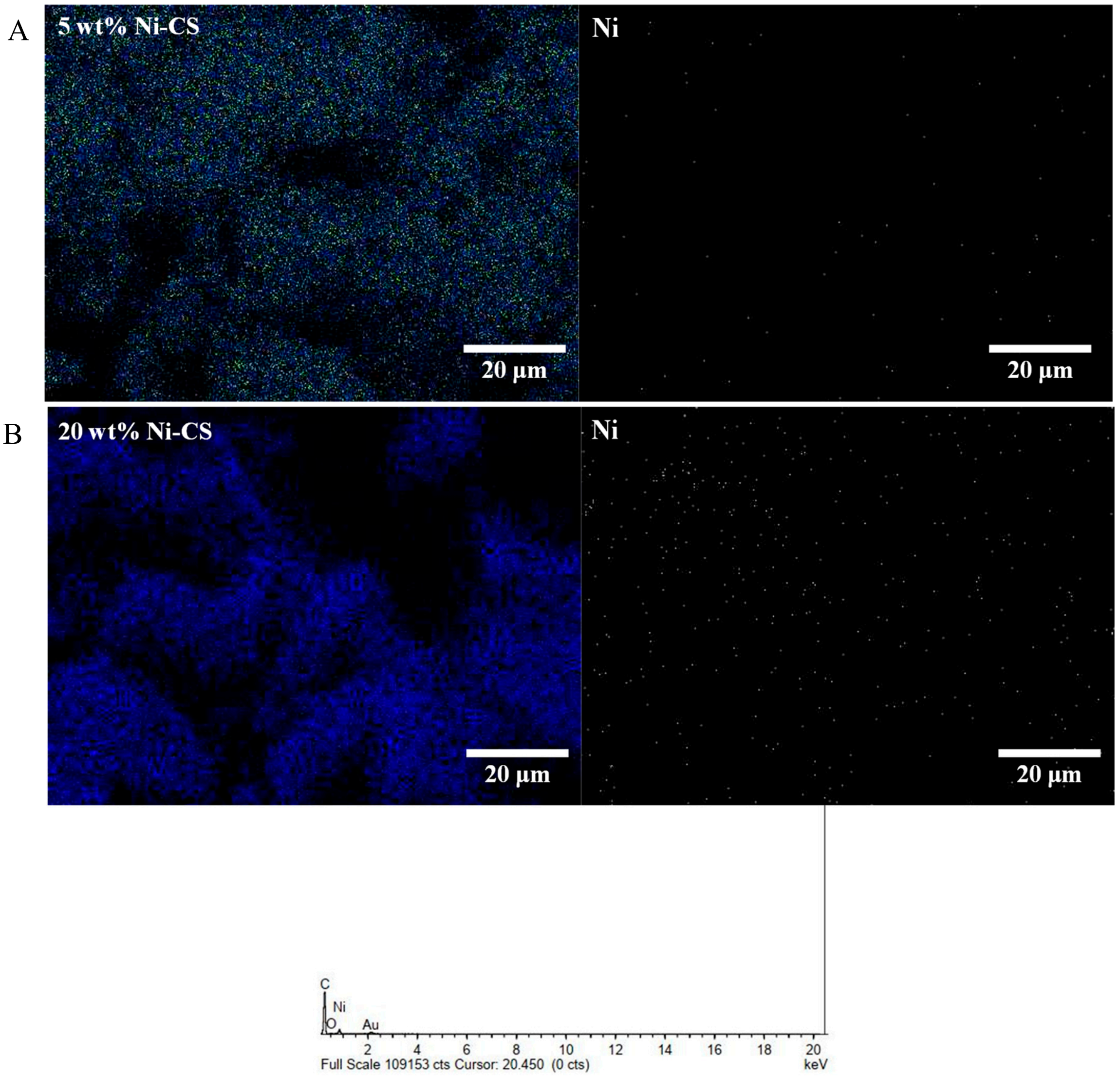

Figure S1. Mapping images and EDX of (A) 5 wt% Ni-CS and (B) 20wt% Ni-CS catalysts.

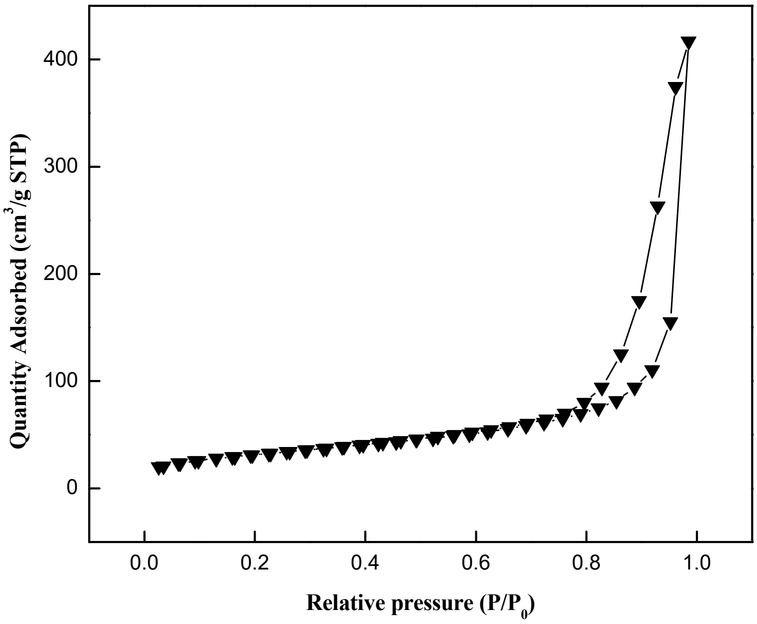

Figure S2. Isotherm linear plot CS from BET analysis.

Table S1: Textural properties of CS.

Sample	S_{BET} (m^2/g)	V_{Total} (cm^3/g)	D_{Pore} (nm)
CS	113.54	0.646	4.41

Table S1 presents the textural properties of CS analysed from BET and BJH analyses. The surface area for CS showed is 113.54 m^2/g, which is comparable with the commercial carbon black (CB) (Vulcan XC-72, Cabot Corporation, USA), ~200 m^2/g [1]. Isotherm linear plot of the CS represents type IV hysteresis loop, mesoporous materials (**Fig. S2**). This also supported by previous research which showed that candle soot has a mesoporous [2].

1. Khodabakhshi, S.; Fulvio, P.F.; Andreoli, E. Carbon Black Reborn: Structure and Chemistry for Renewable Energy Harnessing. *Carbon N. Y.* **2020**, *162*, 604–649,

doi:10.1016/j.carbon.2020.02.058.

2. Yu, H.; Zhang, D.; Fang, Z.; Xu, S.; Liu, Q.; Hou, H.; Wang, L.; Zhou, Z.; Shao, G.; Yang, W.; et al. N and S Co-Doped Carbon Nanofibers with Embedded Candle Soot and Designed Surface Decoration for Efficient Bifunctional Electrocatalysts. *Electrochim. Acta* **2021**, *380*, 138261, doi:10.1016/j.electacta.2021.138261.
